# Identifying ADHD-Related Abnormal Functional Connectivity with a Graph Convolutional Neural Network

**DOI:** 10.1155/2024/8862647

**Published:** 2024-04-30

**Authors:** Yilin Hu, Junling Ran, Rui Qiao, Jiayang Xu, Congming Tan, Liangliang Hu, Yin Tian

**Affiliations:** ^1^Department of Biomedical Engineering, School of Bioinformatics, Chongqing University of Posts and Telecommunications, Chongqing 400065, China; ^2^College of Computer Science and Technology, ChongQing University of Posts and Telecommunications, ChongQing 400065, China; ^3^West China Institute of Children's Brain and Cognition, Chongqing University of Education, Chongqing 400065, China; ^4^Institute for Advanced Sciences, Chongqing University of Posts and Telecommunications, Chongqing 400065, China; ^5^Guangyang Bay Laboratory, Chongqing Institute for Brain and Intelligence, Chongqing 400064, China

## Abstract

Attention deficit hyperactivity disorder (ADHD) is a common neurodevelopmental disorder that is characterized by inattention, hyperactivity, and impulsivity. The neural mechanisms underlying ADHD remain inadequately understood, and current approaches do not well link neural networks and attention networks within brain networks. Our objective is to investigate the neural mechanisms related to attention and explore neuroimaging biological tags that can be generalized within the attention networks. In this paper, we utilized resting-state functional magnetic resonance imaging data to examine the differential functional connectivity network between ADHD and typically developing individuals. We employed a graph convolutional neural network model to identify individuals with ADHD. After classification, we visualized brain regions with significant contributions to the classification results. Our results suggest that the frontal, temporal, parietal, and cerebellar regions are likely the primary areas of dysfunction in individuals with ADHD. We also explored the relationship between regions of interest and attention networks, as well as the connection between crucial nodes and the distribution of positively and negatively correlated connections. This analysis allowed us to pinpoint the most discriminative brain regions, including the right orbitofrontal gyrus, the left rectus gyrus and bilateral insula, the right inferior temporal gyrus and bilateral transverse temporal gyrus in the temporal region, and the lingual gyrus of the occipital lobe, multiple regions of the basal ganglia and the upper cerebellum. These regions are primarily involved in the attention executive control network and the attention orientation network. Dysfunction in the functional connectivity of these regions may contribute to the underlying causes of ADHD.

## 1. Introduction

Attention deficit hyperactivity disorder (ADHD) is a common neurodevelopmental disorder that is characterized by inattention, hyperactivity, and impulsivity [1]. These symptoms have distinct neural bases [2]. In recent years, the prevalence of ADHD in children has gradually increased, with 5.3%–7.1% of children worldwide affected [3]. The effects of ADHD on children's academic ability, social skills, executive functioning, and mood have been shown in several studies [4, 5]. While some children will experience improvement as adults, 70% of cases will have permanent consequences [6]. Such a high lifetime prevalence has a significant impact on the daily life and development of children, as well as a substantial burden on families and society. Therefore, it is becoming more vital to investigate the cognitive mechanisms underlying this neurodevelopmental disorder.

Since the first successful use of functional magnetic resonance imaging (fMRI) to study how the human brain works [7]. An increasing number of studies [8, 9] have used the functional changes observed by fMRI to explore different brain disorders. Since fMRI is sensitive to the whole brain and shows the detailed anatomy of the brain with high spatial resolution [10], it has been widely used in neuro-clinical applications. Resting-state fMRI (rs-fMRI) has become an important tool for the early diagnosis and prognosis of ADHD because it does not require subjects to do complicated tasks and usually only needs them to rest with their eyes open or closed.

Since machine learning and deep learning are effective in various fields, researchers are gradually applying them to the study of fMRI to extract relevant feature information. Machine learning techniques, such as hyperplane-based SVM [11] and decision trees [12], are suitable for 1D features or highly integrated features. With the advent of neural networks in image processing, deep learning models can predict neuroimaging biomarkers more accurately than conventional machine learning techniques [13]. Neural networks that can be utilized with regular grid data, such as convolutional neural networks, deep confidence neural networks, and deep neural networks, have gained prominence. Based on the convolution theorem [14], which states that the convolution in the graph's spatial domain is equal to the inverse Fourier transform of the multiplication in the graph's spectral domain [15], researchers have developed a graph convolutional neural network (GCN) to investigate irregular graph information in the spectral domain. In recent years, there has been increased interest in building GCN models to study the brain connectome [16, 17]. More and more researchers have utilized graph convolutional networks to detect network abnormalities in the brain under various mental health conditions and have achieved remarkable results for both the classification and prediction of neurological diseases. For example, in the field of Alzheimer's disease (AD) research, a functional connectivity-based GCN framework for early prediction of AD [18], a new framework based on multiscale augmented GCN for detecting mild cognitive impairment (MCI) achieved a classification accuracy of 90.39% on the ADNI database [19], sample-weight and feature-weight based on GCN have also made breakthroughs in classification performance and interpretability [20], and there is a structural MRI-based multirelational GCN for AD diagnosis by learning multirelational perceptual representations of brain regions [21]. In the field of ADHD research, there is population-based learning of contrastive functional connectivity graphs for ADHD classification verified to be superior on various metrics [22], and there is a dynamic GCN framework revealing new insights into connectome dysfunction in ADHD [23]. In the others, GCN based on similarity perception and adaptive correction have obtained good results in predicting severe memory problems and MCI [24]. The graph structure data in this paper consists of nodes (brain regions) and edges between nodes (functional connectivity). It is not a 2D grid but indicates whether two brain regions share information. This suggests that, compared with traditional convolutional neural networks, GCN can effectively reflect the complex topological structure of the functional brain network and accurately capture the spatial dependence relationships. Therefore, a GCN model may be a valuable tool for distinguishing individuals with ADHD from typically developing (TD) individuals.

Additional research indicates that functional connectivity may be a significant factor in identifying individuals with ADHD from TD individuals [25]. In this study, rs-fMRI data were utilized to investigate the functional connectivity network in individuals with ADHD and TD individuals. Given the high-dimensional nature of fMRI data, feature selection was necessary before data classification to reduce its high dimensionality [26]. The differences in functional connectivity were input into a GCN to identify individuals with ADHD. After classification, brain regions with significant contributions to classification were visualized based on the results. Furthermore, we explored the relationship between classification features and attention-related regions, as well as the connection between important nodes and the distribution of positively and negatively correlated connections. This exploration allowed us to identify the most discriminative brain regions. These investigations help pinpoint ADHD-related brain regions and the role of attention-related regions, providing a foundation for aiding in the diagnosis and treatment of ADHD.

We propose a GCN architecture and explore the attention-related brain regions to offer a reasonable explanation for the neural mechanisms of ADHD. The major contributions of our study are summarized as follows: (1) Our GCN model achieves cross-subject and cross-site classification of ADHD, outperforming traditional machine learning and other neural networks, particularly in the context of the typical graphical data structure of functional connectivity. (2) In feature selection, we choose differential mean values in ADHD and TD individuals as classification features. This approach not only reduces the high-dimensional characteristics of fMRI data but also enhances classification accuracy for the distinctions between ADHD and TD individuals. (3) Through classification visualization, we can identify the brain regions with the most significant contributions. Combining this information with prior knowledge of attention networks, we can visualize the most discriminative brain regions related to the attention network, providing a theoretical foundation for the diagnosis and treatment of ADHD patients.

## 2. Materials and Methods

### 2.1. Data Acquisition

Data used in the preparation of this paper were obtained from the ADHD-200 public database. The rs-fMRI data of the ADHD-200 database contains data on 947 individuals from eight institutions, of whom 362 have ADHD and 585 do not. At least one rs-fMRI scan, a T1-weighted structural scan, and data on several behavioral characteristics were provided for each subject (age, gender, hand-use habits, and, for most subjects, one or more 1Q scores). In the dataset, subjects were categorized as healthy controls, ADHD-Type C, and ADHD-Type I. Due to uneven data categories (e.g., all normal subjects or only a few ADHD subjects) and incomplete phenotypic information of subjects at some sites, data from Peking University (PK), the Kennedy Krieger Institute (KKI), the New York University Child Study Center (NYU), and Oregon Health and Science University (OHSU) were selected for this paper. Following preprocessing, incomplete or corrupt data were eliminated, leaving 460 subjects.

### 2.2. Preprocessing of rs-fMRI

The raw rs-fMRI data were preprocessed using the data processing and analysis for the brain imaging toolbox [27]. To eliminate the effect of magnetic saturation at the beginning of the scan, the first 10 time points were removed before preprocessing. The preprocessing steps for fMRI were as follows: (1) slice-timing correction to realign 3D brain functional images at each time point; (2) space registration to realign the neural images of each subject with the first data after the removal of 10 time points. We used the Friston 24-parameter model for head motion correction. This is a robust model of head movement that combines six standard head motion parameters for spatial transformation (rigid body model) to perform head motion regression. Afterward, we combined the structural images for segmentation and mapped the functional phases to the MNI standard space by spatial normalization, excluding subjects with translational movements greater than 2 mm and rotational movements greater than 2°. We calculated the mean frame-wise displacement (mean-FD Jackson) for each subject to assess head motion, removing subjects with FD greater than 0.5 mm to ensure data quality and reliability. This step did not eliminate the effects of head motion completely, and head motion covariate control was performed in subsequent group analyses to effectively control head motion noise and eliminate head motion artifacts; (3) normalization and resampling voxel of 3 × 3 × 3 mm^3^. The functional image data and structural item data were realigned, then the T1 structural image data were segmented into white matter, gray matter, and cerebrospinal fluid, and the gray matter density map was applied to the resting state functional image to complete the normalization; (4) smooth, in order to improve the signal quality, using a Gaussian spatial filter with a 6-mm full width at half maximum; (5) remove covariates, white matter, cerebral fluid, whole brain signal, and 24 head motion parameters were removed as covariates in order to improve signal quality; (6) detrend and filter, fMRI signal belonged to low-frequency signal, band-pass filtering (0.001–0.1 Hz) was performed on the signal to remove out-of-band noise.

### 2.3. Construct the Functional Brain Networks

The human brain is a complex system. On the one hand, various brain regions perform distinct functions, while on the other, they collaborate effectively to accomplish more complex cognitive tasks. The current brain atlas divides the cerebral cortex into distinct regions with specific functions. Since there is no consensus on which brain atlas is most accurate in identifying functionally relevant regions of the brain [28]. Thus, the choice of which atlas to use depends on the research objectives, data quality, and considerations of computational complexity and ease of interpretation. We referred to previous studies on functional brain networks, many of which are based on the AAL116 atlas [23, 29], and therefore, we chose the AAL116 brain atlas for ease of comparison with and interpretation of previous research results. In addition, the AAL brain atlas, although with relatively few partitions, reduces the complexity of data processing and analysis and is a simpler and more widely recognized framework. In contrast, high-resolution atlases such as the Schaefer atlas have more partitions, though which also means more complexity in performing data processing and network analysis, requiring more computational resources and time. High-resolution brain atlases are more sensitive to noise, and the fMRI data used in this paper is of average quality, and we eliminated some data during preprocessing, so using Schaefer atlases may not be able to show the expected advantages. In addition, as the number of brain regions increases, it may lead to functional heterogeneity in some regions, i.e., different subregions of the same region may be functionally more different, which will make it more difficult to interpret the results and relate them to other studies. As the aim of this paper is to find the brain regions with large categorical contributions and interpret them at the cognitive level in conjunction with the pathology of attention network theory, to explore their attention-related neural mechanisms, and to find their generalizable imaging biomarkers on the attention network, in order to provide a basis for assisting in the diagnosis and treatment of ADHD. Therefore, we selected the AAL116 brain atlas in this paper. It comprises 90 brain regions in the cerebral cortex and 26 in the cerebellum. These different brain regions are clearly differentiated and collaborate effectively, forming a complex network capable of performing a wide range of cognitive tasks. Functional connectivity is a method used to construct a functional network by leveraging the interdependence of various brain regions. Functional networks consist of nodes and edges. There are three common methods for defining nodes: voxels, regions of interest, and structural template brain regions. In this paper, we use the structured AAL116 template as nodes. Typically, the signal-time or frequency-domain relationship between two corresponding network nodes is used to quantify the functional network edge. Methods like Pearson correlation, partial correlation, mutual information, etc., are frequently employed. This paper utilizes the Pearson correlation method.

**Figure 1:**
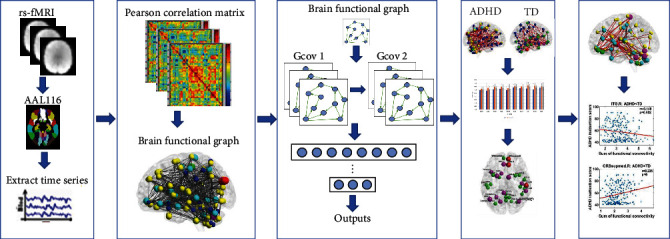
A workflow after data preprocessing. We use differences in functional connectivity as nodal features. After classification, we visualized brain regions with a significant impact on classification outcomes. We also explored the relationship between classification features and regions associated with attention, as well as the impact of important nodes on the distribution of positively and negatively correlated connectivity.

The procedure following data preprocessing is shown in Figure 1. Time signals were extracted using the AAL116 template, and the time series of all voxels within each region were spatially averaged, resulting in data dimensions of (116, *n –* 10), representing 116 brain regions, each with *n –* 10 time points. The connectivity between brain regions was determined by calculating the correlation between time series; a stronger correlation indicated synergy between brain regions. Pearson correlation coefficients were used to construct the functional connectivity matrix of (116, 116) by calculating the correlations between any two brain regions. The correlation coefficient matrix of the functional brain network was then subjected to Fisher *Z* transformation to create the functional brain network. As nodes in graph theory, 116 brain regions were employed, and the correlation coefficients between brain regions served as edges. Functional connectivity has been used as a classification feature in many previous classification studies, and the accuracy of classification based on multisite data in previous studies is low due to the presence of site effect, which may also be related to the small sample size and high dimensionality of fMRI data. Considering the effects of these factors, this study first resampled the data to reduce the effects of heterogeneity of different sampling parameters. Second, the functional connectivity matrix of the subjects was statistically analyzed using different *p*-values, and the connections with large differences obtained from the statistical analysis were used as classification features to reduce the effect of low classification accuracy due to high data latitude. The Regress operation was performed afterward to remove sites as covariates. Ten-fold cross-validation was used to evaluate the classification effect. Since the fMRI data were acquired at four different sites, we then performed a stratified sampling of the data by drawing on the methodology used in a previous study, dividing the data into 10 strata according to the acquisition site [23]. Recent studies have shown that an SMA-based standardization scheme for de-site effects in multicenter big data for brain imaging can help to further improve the accuracy and reliability of multisite studies [30], which is also useful for us in future studies. In this study, we selected connectivity features with differences in mean values for classification. Age, gender, hand-use habits, sites, and six head movement signals were removed as covariates in the two-sample *t*-test to reduce the influence of irrelevant variables on the data. For classification features, we chose connectivity at different *p*-values, ranging from 0.005 to 0.05, with an interval of 0.005.

### 2.4. Construction of Graphs

The graph *G* consists of a vertex *V* and an edge *E* (*V*, *E*), where *V*={*v*_1_, ⋯, *v*_*n*_} is the vertex set and *E*⊆*V* × *V* is the edge set. Let *n* be the number of vertices and *m* be the number of edges. Each graph can be represented by an adjacency matrix *A* of size *n* × *n*, where *A*_*ij*_=1 if there is a connectivity from vertex *v*_*i*_ to *v*_*j*_, otherwise *A*_*ij*_=0. In this paper, we used AAL116 brain regions as a template to extract the average time series of each brain region and calculated the Pearson correlation coefficients between brain regions as edges to construct a functional connectivity network. The functional connectivity network was binarized according to the set threshold to form a binary network, and a GCN model was constructed for graph classification.

Based on what convolution means, the GCN model is a filter built in the Fourier domain. According to the convolution theorem, the convolution equation is as follows:

(1) $$\begin{array}{c} f\times g=F^{-1}\left\{F\left\{f\right\}F\left\{g\right\}\right\}. \end{array}$$

Define its first-order derivative on the graph as follows:

(2) $$\begin{array}{c} f_{\times g}\left(x\right)=f\left(x\right)-f\left(y\right), \end{array}$$

where *x* and *y* are neighboring nodes whose corresponding second-order derivatives, the Laplace operators are defined as follows:

(3) $$\begin{array}{c} \Delta_{\times g}f^{\prime }\left(x\right)=\sum_{y∼x}f\left(x\right)-f\left(y\right). \end{array}$$

Define *D* ∈ (*n* × *n*) as the degree matrix of the adjacency matrix *A* when *i*=*j* and *D*(*i*, *j*) as the degree of the node *d*_*i*_, then the Laplace operator of the graph can be written as *L*=*D* − *A*, and the normalized Laplace operator is shown as follows:

(4) $$\begin{array}{c} L=I_{N}-D^{-\frac{1}{2}}ADD^{-\frac{1}{2}}. \end{array}$$

The Fourier basis of the graph is the eigenvector of the Laplace matrix of *U*=[*u*_1_, ⋯*u*_*n*_], at this time *L*=*UΛU*^*T*^, where *Λ* is the diagonal matrix composed of eigenvalues. At this point, the graph Fourier transform equation and the Fourier inverse transform are as follows:

(5) $$\begin{array}{c} \left\{\begin{array}{c} \text{GF}\left\{X\right\}=U^{T}x\\ \text{IGF}\left\{X\right\}=Ux \end{array}\right.. \end{array}$$

The convolution expression of the graph can be obtained from the Fourier definition equation of Equation (1) and the Fourier transform Equation (5) of the graph:

(6) $$\begin{array}{c} g\times x=U\left(U^{T}gU^{T}x\right), \end{array}$$

where *g* is the filter function, to give the network better local properties during convolution so that it affects only neighboring vertices, *g* is defined as a function of the Laplacian matrix *g*(*L*). Put *U*^*T*^*g* is a function *g*_*θ*_(*Λ*) of the eigenvalue matrix *Λ* of the Laplacian matrix *L* with the parameter *θ*. At this point, the convolution equation is as follows:

(7) $$\begin{array}{c} g_{\theta }\times x=Ug_{\theta }U^{T}x=Ug_{\theta^{\prime }}\left(\Lambda \right)U^{T}x. \end{array}$$

The above equation needs to require the eigenvectors of the Laplace matrix; for the brain function, connected graphs have more vertices and edges, the process of solving the eigenvectors is complicated, the approximation of the filter function according to the Chebyshev polynomial can save that complicated step, when the order *K* of the Chebyshev inequality is taken as 1, the forward transfer to the function of the convolution of the graph can be obtained, the formula is as follows:

(8) $$\begin{array}{c} H^{l+1}=\sigma \left({\tilde{D}}^{-\frac{1}{2}}\tilde{A}{\tilde{D}}^{-\frac{1}{2}}H^{l}W^{l}\right). \end{array}$$

**Figure 2:**
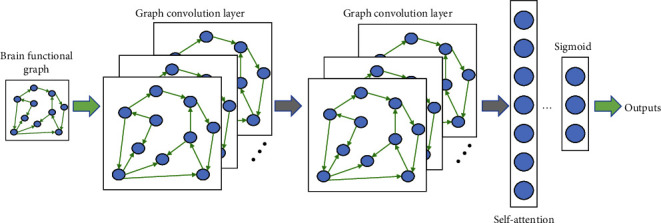
A 2-layer GCN model with a self-attentive pooling mechanism was built in this study and called 2L_AGCN. The number of convolutional kernels is 32 and 16; the pooling parameter is 0.35; the activation function is ReLU; the learning rate is 0.034; and the weight decay is 0.00005.

The structure of GCN is shown in Figure 2, which is a subgraph level GCN that requires graph embedding, converting the connectivity matrix into a connectivity table *DA* to facilitate the construction of the adjacency matrix *A*. *DA* consists of coordinates that represent the connectivity between nodes *i* and *j*. *DA* contains the connectivity of all graphs; for each node, record the node label, which is a different brain region. For each node, the graph embedding process requires a graph index to record the number of graphs it belongs to. This study is a supervised binary classification task, so graph labels are needed. Since the self-attention mechanism plays an important role in improving the performance and interpretability of neural networks [31]. Therefore, a two-layer GCN model with a self-attentive pooling mechanism was built in this study and called 2L_AGCN. The number of convolutional kernels is 32 and 16; the pooling parameter is 0.35; the activation function is ReLU; the learning rate is 0.034; and the weight decay is 0.00005.

## 3. Results

### 3.1. Classification Accuracy

**Figure 3:**
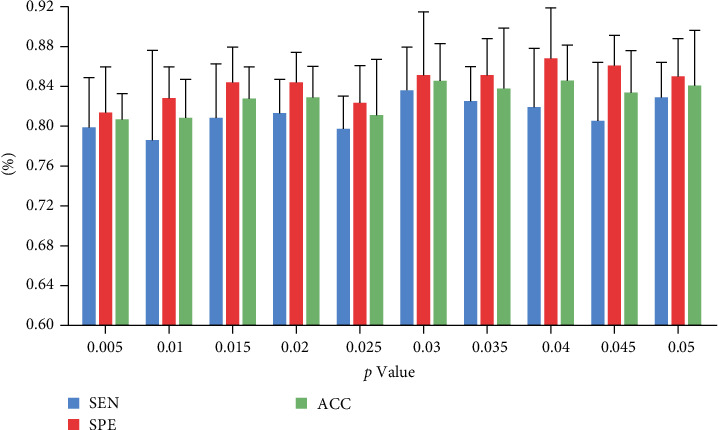
Variation of classification accuracy with *p* value. The best classification accuracy was achieved at *p*=0.03 with SEN = 83.64% ± 4.36%, SPE = 85.12% ± 5.95%, and ACC = 84.49% ± 3.53%.

In this study, sensitivity (SEN), specificity (SPE), and accuracy (ACC) were used as measures of classification accuracy [23]. A one-tailed, two-sample *t*-test was done on the data to investigate the functional connectivity in the case of different execution spaces *p* (0.005–0.05) as input. The classification accuracy obtained with 10-fold cross-validation is shown in Figure 3, where the classification accuracy first increases with increasing *p*-value and then gradually plateaus. With *p*=0.005, the classification accuracy is 80%. The best classification accuracy is achieved at *p*=0.03, with *Ss* = 83.64% ± 4.36%, *Sc* = 85.12% ± 5.95%, and *Gr* = 84.49% ± 3.53%. To provide a basis for comparison, we adopt two baselines: the SVM using the radial basis function kernel and a traditional GCN model without attention mechanisms. The classification results are shown in Table 1. SVM has the highest classification accuracy when *p*=0.045, and GCN has the best classification accuracy when *p*=0.035. The results show the effectiveness of our proposed GCN model with an attention mechanism. We used a leave-one-site-out cross-validation procedure to assess the GCN model's ability to classify data from various sites. This procedure excluded data from one site from the training process, trained the model iteratively using data from three other sites, and evaluated the model using data from the excluded site. The study selected information from PK, KKI, NYU, and OHSU.  Table 2 displays the results of leave-one-site-out cross-validation.

### 3.2. Visualization of Functional Connectivity

Different functional connectivity at *p*=0.03 was used as input to achieve the highest degree of precision. The total number of brain network connectivity was 1,110, with 599 connectivity in the ADHD > TD group and 511 connectivity in the ADHD < TD group, as visualized by the brain network of differentially functional connectivity.

**Figure 4:**
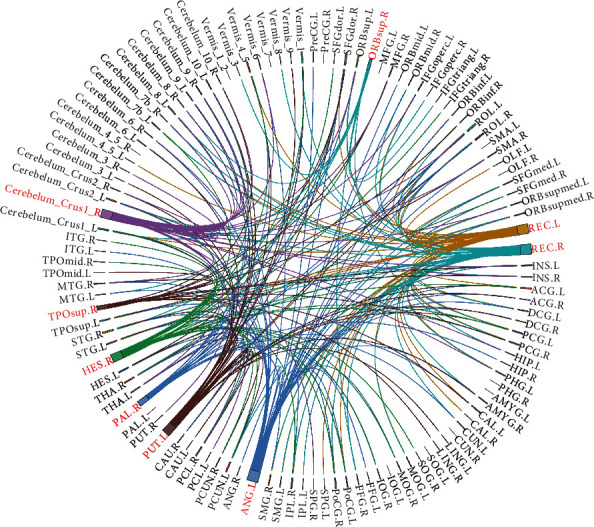
The coordinates represent different brain regions, and the lines represent the functional connectivity between each brain region. We visualized several brain regions with a higher nodal degree and marked them in red.

Some regions of the frontal, parietal, and cerebellar lobes of the brain had higher nodal degrees; Figure 4 shows the brain regions with the highest nodal degree. The region of the brain with the highest nodal degree is the left angular gyrus in the parietal lobe. Additionally, the nodal degrees in the gyrus rectus, the right superior frontal gyrus, the medial orbital, the right Heschl's gyrus, the left lenticular nucleus, the putamen, and the right superior cerebellar area 1 are significant.

### 3.3. Visualization of ADHD Classification Contribution Regions

**Figure 5:**
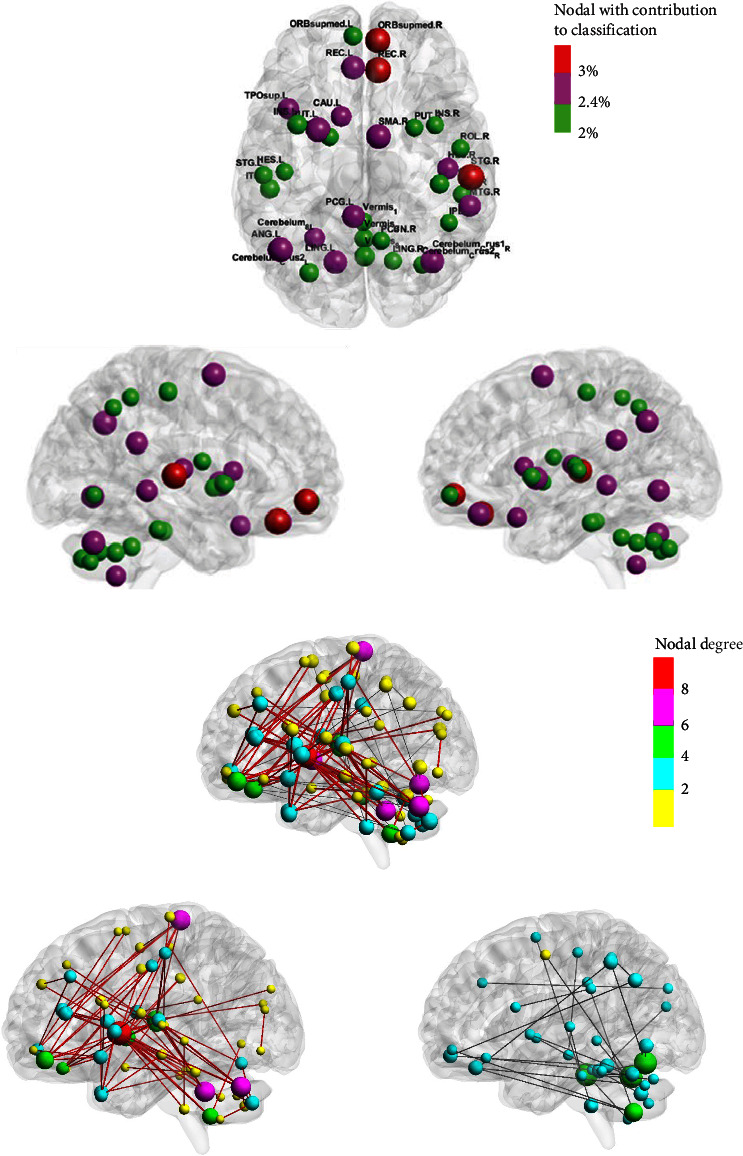
Visualization of brain regions: (a) brain regions with high contribution to classification (>2%), those with a classification contribution of 3% are red nodes, those with a contribution of 2.4% and 3% are purple nodes, and those with a contribution of 2.4% are green nodes; (b) the functional connectivity with significant correlations (uncorrected, *p*  < 0.05). The nodes indicate different brain regions, the red lines indicate positively correlated connectivity, and the black lines indicate negatively correlated connectivity.

The contribution of each brain region to the classification was calculated by superimposing the weight values of the functional connectivity connected to each node in the classification. The size of the nodes varied with the percentage of classification contribution; a total of 34 nodes with a large contribution (>2%) to the visualization are shown in Figure 5(a), and the overall classification contribution of these nodes is greater than 80%. REC.L/R indicates left and right rectus gyrus, STG.R indicates right superior temporal gyrus, ORBsup.R indicates right superior frontal gyrus, orbital part, TPOsup.L indicates left superior temporal gyrus, CAU.L indicates left caudate nucleus, PUT.L indicates left lenticular nucleus, putamen, SMA.R indicates right supplementary motor area, MTG.R indicates right middle temporal gyrus, Cerebelum_crus1_R indicates right cerebellar area 1, PCG.L indicates left posterior cingulate gyrus, ANG.L indicates left angular gyrus, LING.L indicates left lingual gyrus. Nodes with a classification contribution of 3% are highlighted in red, nodes with contributions ranging from 2.4% to 3% are marked in purple, and nodes with a contribution of 2.4% are represented in green. There are three red nodes, all located in the right side of the brain, namely the gyrus rectus, superior temporal gyrus, and superior frontal gyrus, as well as the orbital part; 12 purple nodes are distributed in the frontal, temporal, and parietal lobes; and 19 green nodes are mainly distributed in the cerebellum and parts of the temporal lobe. Their specific categorical contributions are shown in Table 3. Prefrontal regions (rectus gyrus and superior orbital frontal gyrus), the striatum, the temporal lobe (superior temporal gyrus, middle temporal gyrus, and inferior temporal gyrus), some regions of the parietal lobe, and some regions of the cerebellum are the regions with large classification contributions. Seven of these cerebellar regions indicate that cerebellar-related connectivity is also important for differentiating ADHD from TD individuals. In recent years, previous studies have explored the possible role of different brain regions in ADHD pathology. In addition, a range of cognitive deficits, including symptoms of impaired working memory, have been observed in ADHD individuals due to abnormal development of cerebellar structures [32]. More gray matter volumes were observed in studies of adults and adolescents with ADHD, which further indicated that ADHD was related to cerebellum dysfunction [32]. In conclusion, our results are consistent with existing investigations and build on previous results indicating that the frontal, temporal, parietal, and cerebellar regions may be the main areas of dysfunction in ADHD individuals.

### 3.4. ADHD Difference Network and Attention Correlation Analysis

Previous theories have shown that ADHD is strongly associated with abnormalities in the attentional network, and the purpose of this paper was to explore the neural mechanisms underlying the association between ADHD and attention. To better understand the relationship between attention deficit and ADHD symptom severity, we calculated functional connectivity between all pairs of brain regions and investigated their correlation with ADHD-RS ADHD inattention score in the ADHD group. The sum of the *Z* values of functional connectivity of all nodes was correlated with the inattention score to explore which brain regions may cause attention deficits. Nodes had both enhanced and weakened connectivity. To avoid cancelations between enhanced and weakened connectivity, the absolute values of *Z*-values of functional connectivity were weighted and summed at each node according to the methods of ADHD > TD and ADHD < TD, then correlation analysis was performed with the subjects' inattention score. The higher the ADHD inattention score, the poorer the attentional concentration ability. The presence of a positive correlation indicates that the stronger the functional connectivity, the more significant the degree of attentional deficits. A negative correlation indicates that as the strength of functional connectivity decreases, the degree of attentional deficits becomes more significant.

The functional connectivity with significant correlations (uncorrected, *p* < 0.05) is shown in Figure 5(b). Our purpose is to screen the significant regions and the regions that have a large contribution to the previous classification to get the most discriminative brain regions. However, there were fewer significant brain regions after FDR correction, which did not have statistical significance. Therefore, we did not correct for multiple testing in this exploratory study. All statistical data were analyzed at a 0.05 level of significance. The uncorrected results, combined with previous studies, may reveal the relationship between ADHD and attention deficit, which is cognitively interpretable. Since this paper is an exploratory study on cognition, uncorrected *p*-value has been proven to be useful for exploratory analysis. Some studies have explored the relationship between early-onset obsessive–compulsive disorder and local gyrification index through uncorrected *p*-value [33]. There are also some studies without multiple comparison corrections [34, 35], and some studies report both corrected and uncorrected results [36–38]. The larger the node degree, the larger the nodes in the graph, while the node ranges are marked with different colors. Figure 5(b) shows all connectivity significantly related with attention: 129 in total, with 97 positive and 32 negative correlations. Important nodes of ADHD individuals are in anterior brain regions (basal ganglia, prefrontal cortex, thalamus), several vermes, and inferior regions of the cerebellum. Most of the positively correlated connectivity is concentrated in the prefrontal to parietal and temporal connectivity. The more inattentive the subjects are, the stronger the functional connectivity in the prefrontal lobe, which may be the reason for the inattention of children with ADHD. While the negatively correlated connectivity is mainly from the frontal–parietal lobe to the cerebellum, indicating that the more inattentive the subjects are, the weaker the connectivity is. It should be noted that the exploratory analyses were not corrected for multiple testing. Therefore, the results of these analyses must be replicated in future confirmatory studies.

ADHD is characterized by attention deficits. Multiple studies on ADHD have revealed abnormalities in various attention-related brain regions and networks. Qualitative analyses of rs-fMRI data consistently show significant differences in sensory areas, including the anterior cingulate gyrus, prefrontal cortex, putamen nucleus, occipital cortex, and temporal cortex in ADHD patients [39, 40]. Additionally, low-frequency oscillations in the cerebellum and thalamus of individuals with ADHD differ significantly from those of TD individuals [41]. Abnormalities in internal functional connectivity are observed in the default network, somatosensory network, and dorsal attention network, as well as abnormal internetwork functional connectivity in the default network and ventral attention network, were present in children with ADHD and were positively correlated with ADHD symptoms [42]. The brain regions associated with attention that are discussed here were all implicated in ADHD, and their abnormalities align with the findings presented in this study.

In this study, there is a correlation between the strength of functional connectivity and attention in ADHD patients. Putamen, pallidum, thalamus, the left cerebellar area 1, the right paracentral lobule, vermis 1, vermis 6, the left superior frontal gyrus, orbital part, gyrus rectus, the right Heschl's gyrus, and the inferior temporal gyrus have the highest nodal degrees. In addition to these regions comprising the anterior cingulate and paracingulate gyri, the insula and several cerebellar regions are involved. It is possible that damage to the structure or function of these attention-related regions can result in abnormalities in the functional information transmission network. A positive correlation indicates that as functional connectivity strength increases, the severity of attention deficit also increases. Previous studies indicated that the posterior cerebellum is involved in visuospatial function and attentional orienting, whereas the left cerebellar areas 1 and 2 were involved in spatial attentional control [43]. Children with ADHD have enhanced connectivity in left cerebellar areas 1 and 2 and more active attentional-orienting functions, which may be related to the fact that they are more easily drawn to the outside world.

### 3.5. Categorical Contribution Region and Attention Correlation Analysis

**Figure 6:**
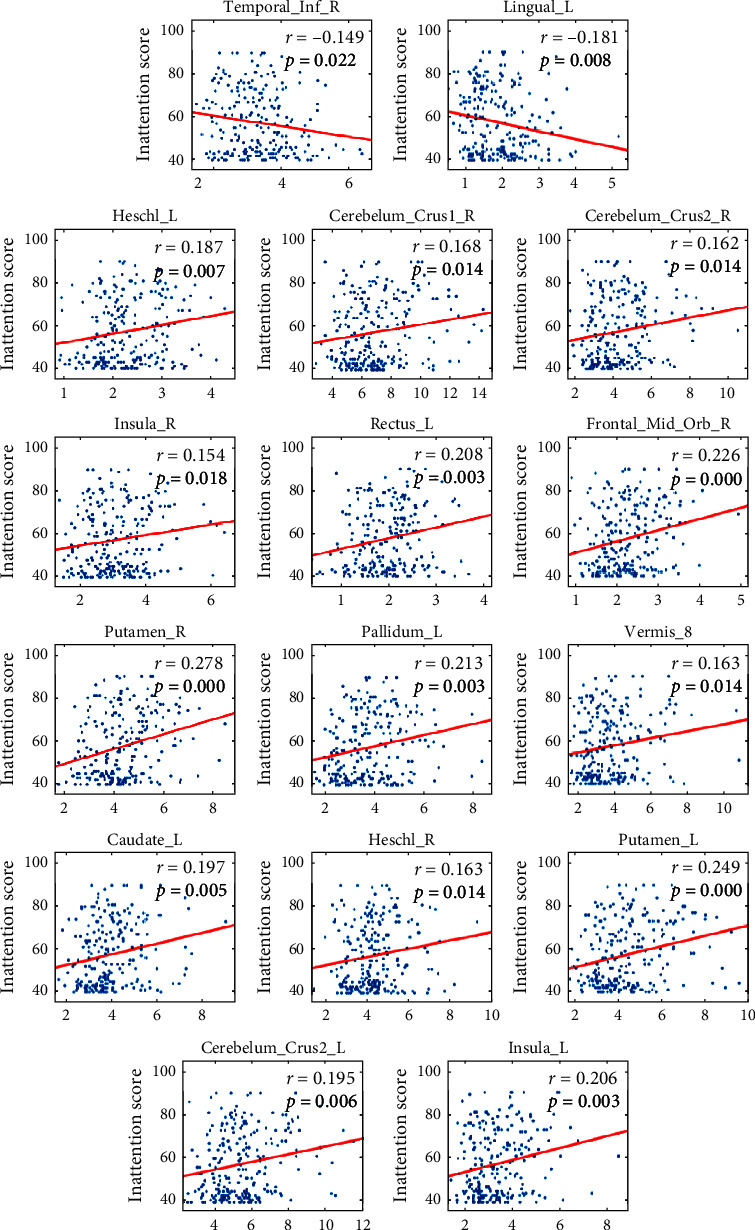
Correlation between functional connectivity and inattention score. Here, *p* values are FDR corrected. There are two negative correlations in the ADHD < TD group and 14 positive correlations in the ADHD > TD group.

Through the above analysis, we visualized a total of 16 brain regions, which are not only the regions with greater categorical contributions but also the regions significantly associated with inattention scores (*p* < 0.05). There are two negative correlations in the ADHD < TD group and 14 positive correlations in the ADHD > TD group, which are shown in Figure 6. The most discriminative brain regions include the right superior frontal gyrus, orbital part, left gyrus rectus and insula in the frontal lobe, right inferior temporal gyrus and Heschl's gyrus in the temporal lobe region, lingual gyrus in the occipital lobe, left caudate nucleus, right putamen, and left pallidum, as well as the superior cerebellum and vermis 8, which are mostly involved in executive control of attention and attentional orienting networks according to Posner's attention model [44].

## 4. Discussion

In this paper, rs-fMRI data were used to investigate the functional connectivity network of ADHD and TD individuals, and we visualized the brain regions with high classification contributions. Our investigation revealed that the frontal, temporal, parietal, and cerebellar regions contributed significantly to the classification of ADHD. Additionally, to explore the relationship between classification features and attention-related regions, we visualized the brain regions associated with attention networks. Finally, we filtered out a total of 16 brain regions, including the right superior frontal gyrus, the left direct frontal gyrus, and other brain regions. These regions are primarily involved in the executive control of attention and the attention orientation network. These investigations can help identify ADHD-related brain regions and the role of attention-related regions, providing a basis to assist in the diagnosis and treatment of ADHD.

We classified the data from PK, KKI, NYU, and OHSU, and we obtained an accuracy of 84.49% ± 3.53%. Previous studies used data from three different sites and investigated which regions, including the prefrontal, cingulate, and visual cortexes, can best distinguish between healthy controls and different subtypes of ADHD (inattentive, hyperactive, and mixed ADHD) [45]. With an accuracy of 90% for comparisons, a fully connected cascade artificial neural network was used to distinguish ADHD and TD individuals. Connectivity between the frontal lobes and cerebellum was the defining characteristic [46]. These studies had relatively high classification accuracy to distinguish ADHD from TD individuals and identified problems in multiple regions of the brain related to attention.

We identified the input features of functional connectivity differences and obtained brain regions with a greater contribution to classification. Our results showed that specific regions in the frontal, temporal, parietal, and cerebellar lobes had higher nodal degrees. The results were in line with several previous studies, but there were also some differences. Research indicates that the frontal and cerebellar regions [47], as well as the prefrontal, cingulate, and visual cortex [45], were the most discriminative. In distinguishing ADHD from TD individuals, the temporal lobe, cingulate gyrus, and ventral lateral prefrontal cortex were crucial regions [39]. Local connectivity and regional homogeneity calculations could effectively distinguish the ADHD group from the control group. The prefrontal cortex, anterior cingulate gyrus, and cerebellum were discovered to be the brain regions with the greatest degree of differentiation [48]. The functional connectivity between the frontal and cerebellar regions appeared to be a robust candidate for the distinction between ADHD and TD individuals.

Our findings suggest that ADHD is associated with abnormalities in multiple brain regions, indicating that it is not caused by a single brain region but by issues in how the brain's attention networks connect. These findings align with previous studies [47]. Several regions, such as the right prefrontal cortex, cerebellum, and caudate nucleus, have shown structural abnormalities [49]. Using a local coherence index, the rs-fMRI of boys with ADHD was examined in a study. It was discovered that local coherence in the frontal–striatal-cerebellar pathway decreased, whereas it increased in the occipital lobe [50]. ADHD patients exhibited enhanced functional connectivity in the anterior cingulate gyrus and dorsal frontal lobe [51]. ADHD was associated with decreased low-frequency amplitudes in the right inferior frontal gyrus cortex, left sensorimotor cortex, and both cerebella and increased low-frequency amplitudes in the right anterior cingulate cortex and bilateral brainstem, indicating that changes in spontaneous neural activity in these brain regions may be related to the pathophysiological mechanisms of ADHD [52]. Researchers studying the inhibition-related modulation of the frontoparietal network in children with ADHD found that it predicted cognitive control and inattention symptoms in children. These studies indicated that ADHD is a closely related psychiatric disorder [53].

In our results, most of the positively correlated connectivity is concentrated in the prefrontal to parietal and temporal connectivity, while the negatively correlated connectivity is mainly from the frontal–parietal lobe to the cerebellum. We obtained many attention-related brain regions through visualization, which indicates ADHD is a mental disorder closely associated with attention. Most of the brain regions are involved in the attentional orienting networks and the attentional executive control networks. A core symptom of ADHD is the inability to ignore external stimuli, which is thought to result from abnormalities in the functional connectivity of the dorsal attentional network [54]. The intraparietal sulcus and the frontal eye area are vital components of the dorsal attention network, while the parietal lobe plays a crucial role in attentional dissociation. In addition, the right posterior cerebellar lobe, middle temporal gyrus, and inferior temporal gyrus are important dorsal network components. The ventral attentional network consists of the right temporoparietal connectivity and ventral frontal cortex, and it redirects attention to stimuli [54]. Correlation analysis between the sum of connectivity strengths of contributing brain regions and attention deficits revealed regions such as the right superior orbital frontal gyrus, the left rectus gyrus, and bilateral insula in multiple regions of the prefrontal lobe, the right inferior temporal gyrus and bilateral transverse temporal gyrus in temporal lobe regions, the lingual gyrus in the occipital lobe, and the left caudate nucleus [41]. This is consistent with the presence of multiple functional connectivity abnormalities in parietal and temporal brain regions in the present study and indicates that functional connectivity in parietal and temporal brain regions can be used to identify ADHD and TD individuals.

There were some limitations to this research. Single static features were used for classification and identification in this study; multiple features were not used for classification studies. This study focused solely on differences in the strength of functional connectivity in ADHD. The flow of information was not identified. In addition, existing research has indicated that extracting connectivity features from multiple brain atlases improves the diagnostic precision of brain disorders [55]. The current approach to identifying the best applicable brain atlases for different scenarios is mainly data-driven [28], i.e., instead of assuming a priori what atlases would be appropriate to use, atlases with different levels of granularity are to be used, and their performances are to be compared in terms of recognizing signals of interest. In the future, we will conduct an in-depth study of ADHD based on multiple brain maps and multiple features, as well as the information flow of ADHD, to explore the etiology of ADHD and improve diagnostic and treatment approaches.

## 5. Conclusions

In this paper, a GCN model was developed to classify the functional connectivity differences between ADHD and TD individuals. It was investigated that functional connectivity differences could be used to identify ADHD and TD individuals with high classification accuracy. In addition, we visualized the functional connectivity network and the regions that contributed the most to the classification, investigated the correlation between the features of the attention-related network regions and the inattention scores, and filtered the most discriminative regions that contributed the most to the classification and were significantly associated with attention. The results revealed that the attention-related regions were primarily located in the prefrontal and cerebellar regions of the brain and that these regions helped identify ADHD individuals. There are problems in the default mode network, the ventral attention network, the executive control network, and other attention-related networks, especially in several dorsal attention network regions. Disruptions in the functional connectivity of these regions could be the root cause of ADHD disorders.

## Figures and Tables

**Table 1:** Classification performance comparison (%).

	*Ss* (mean ± std)	*Sc* (mean ± std)	*Gr* (mean ± std)
SVM	72.76 ± 6.17	71.72 ± 5.84	72.24 ± 5.27
GCN	80.21 ± 5.86	78.45 ± 5.62	79.43 ± 4.14
Ours	83.64 ± 4.36	85.12 ± 5.95	84.49 ± 3.53

**Table 2:** Classification performance (%) of leave-one-site-out cross-validation.

	*Ss* (mean ± std)	*Sc* (mean ± std)	*Gr* (mean ± std)
PK	57.45 ± 6.72	62.07 ± 6.43	58.76 ± 5.65
KKI	55.86 ± 7.62	51.22 ± 5.44	53.97 ± 6.58
NYU	64.24 ± 5.85	60.90 ± 8.53	62.17 ± 6.38
OHSU	56.55 ± 7.43	52.24 ± 9.31	54.86 ± 6.60

**Table 3:** Classification of brain regions with a high contribution.

Brain area	Contribution rate (%)	Brain area	Contribution rate (%)
Rectus_R	3.13	Cerebelum_Crus2_L	2.26
Temporal_Sup_R	3.12	Temporal_Inf_L	2.25
Frontal_Sup_Orb_R,	3.00	Vermis_9	2.25
Putamen_L	2.96	Postcentral_R	2.20
Angular_L	2.93	Frontal_Sup_Orb_L	2.16
Supramarginal_R	2.92	Temporal_Inf_R	2.16
Cerebelum_Crus1_R	2.84	Vermis_1	2.15
Rectus_L	2.80	Pallidum_L	2.09
Lingual_L	2.79	Rolandic_Oper_R	2.07
Paracentral_L	2.71	Temporal_Sup_L	2.07
Temporal_Mid_R	2.58	Lingual_R	2.06
Heschl_R	2.53	Precuneus_R	2.05
Temporal_pole_sup_L	2.48	Putamen_R	2.05
Caudate_L	2.41	Insula_R	2.03
Cerebelum_8_L	2.40	Cerebelum_Crus2_R	2.02
Vermis_8	2.37	Pallidum_L	2.01
Insula_L	2.30	Heschl_L	2.00

## Data Availability

All MRI data used in this study are publicly available at the International Neuroimaging Data-sharing Initiative website (http://preprocessed-connectomes-project.org/adhd200/).
